# Reverse Takotsubo Cardiomyopathy Following COVISHIELD Vaccination: A Rare Case of an Anaphylactic Reaction

**DOI:** 10.7759/cureus.43257

**Published:** 2023-08-10

**Authors:** Mandar M Shah, Tapan Kumar, Aparna Prajapati, Zaid Nafe

**Affiliations:** 1 Department of Cardiology, Tata Main Hospital, Jamshedpur, IND; 2 Department of Gastroenterology, Tata Main Hospital, Jamshedpur, IND

**Keywords:** takotsubo cardiomyopathy (ttc), reverse ttc, covishield, covid-19 vaccine, anaphylactic reaction

## Abstract

This case report describes a 47-year-old female healthcare worker who developed reverse takotsubo cardiomyopathy (TTC) following the administration of the COVISHIELD COVID-19 vaccine. Within minutes of receiving the vaccine, she experienced acute shortness of breath, nausea, and restlessness, along with a significant drop in blood pressure. She was diagnosed with an anaphylactic reaction and promptly treated with adrenaline and fluids. In the intensive cardiac care unit, she exhibited hypotension, had vision loss, and developed pulmonary edema. Further evaluation revealed abnormal ECG findings, elevated troponin levels, and reduced left ventricular ejection fraction (LVEF). Coronary angiography ruled out obstructive coronary artery disease. The patient gradually improved over several days and was discharged with a recovered left ventricular function. This case highlights the occurrence of TTC triggered by anaphylaxis to the COVID-19 vaccine and emphasizes the need for preparedness to manage such emergencies in vaccination centers.

## Introduction

Takotsubo cardiomyopathy (TTC), also known as “stress cardiomyopathy” or “broken heart syndrome,” is a temporary and reversible heart condition characterized by clinical presentation, ECG, cardiac enzymes, and echocardiography mimicking ACS but with normal coronaries, which typically occurs following intense emotional and mental stress [1], with resultant excessive catecholamine surge being identified as the most plausible mechanism [2]. However, this condition could sometimes be triggered by a reaction to a drug [3-5] and/or iatrogenic or exogenous catecholamine administration [6]. Till now, no case has been reported following administration of a vaccine. Here, we describe a case of a 47-year-old female who developed reverse TTC following COVISHIELD vaccination [7]. COVISHIELD™ [previously known as ChAdOx1 nCoV-19 coronavirus vaccine (recombinant)] consists of a recombinant, replication-deficient simian adenovirus that encodes the SARS-CoV-2 spike protein with a tissue plasminogen activator (tPA) leader sequence. The spike protein antigen gene is expressed once the recombinant adenovirus enters the cells of a vaccinated individual. The recombinant adenovirus is propagated and manufactured using the T-REx-293 permissive host cell line that was derived from a HEK-293 cell line [8].

## Case presentation

A 47-year-old female, a healthcare worker (nursing administrator) with no prior comorbidities, received a COVID-19 vaccine, COVISHIELD. Within five minutes of receiving the dose, she developed acute shortness of breath, nausea, severe uneasiness, and restlessness. A quick examination in the vaccination center revealed that she had a thready pulse and systolic blood pressure of 70 mmHg. Air entry was markedly reduced bilaterally. A bedside clinical diagnosis of an anaphylactic reaction to the vaccine was made, and she was administered intravenous adrenaline and intravenous (IV) normal saline. She was immediately shifted to the intensive cardiac care unit (ICCU). When received in ICCU, she was extremely agitated, gasping for breath, and tossing in bed. She complained of complete loss of vision. She did not have a recordable pulse or BP. Extremities were cold and clammy. Air entry was absent bilaterally. She was immediately given two additional doses of adrenaline injection at a five-minute interval because of an anaphylactic reaction. IV fluids were continued. A quick bedside 2D echocardiography (ECHO) screening revealed normal left ventricular ejection fraction (LVEF). Soon her restlessness reduced, her vision was restored, and her pulse became palpable, but she developed intractable hypotension and required inotropic support (noradrenaline infusion). Within a few hours, she developed features of pulmonary edema (orthopnea with bilateral basal crepitations). She also complained of chest pain. Her oxygen saturation dropped to 88% even with 5 L of oxygen and required high-flow nasal oxygen support.

**Figure 1:**
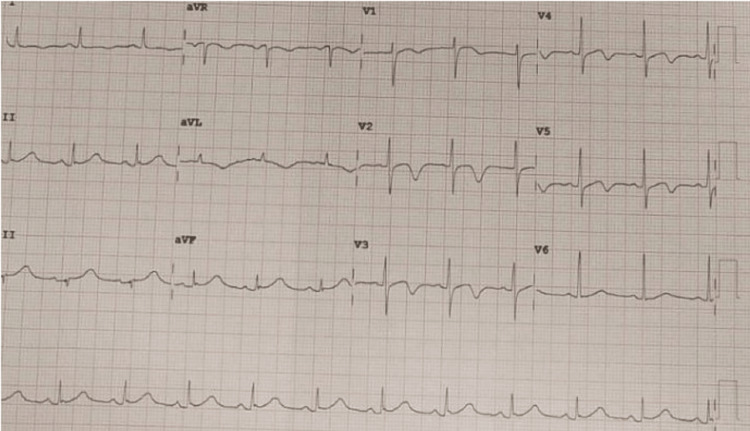
ECG showing ST-segment depression with T-wave inversion in chest leads

ECG showed QT prolongation with ST-segment depression in chest leads (Figure 1).

**Figure 2:**
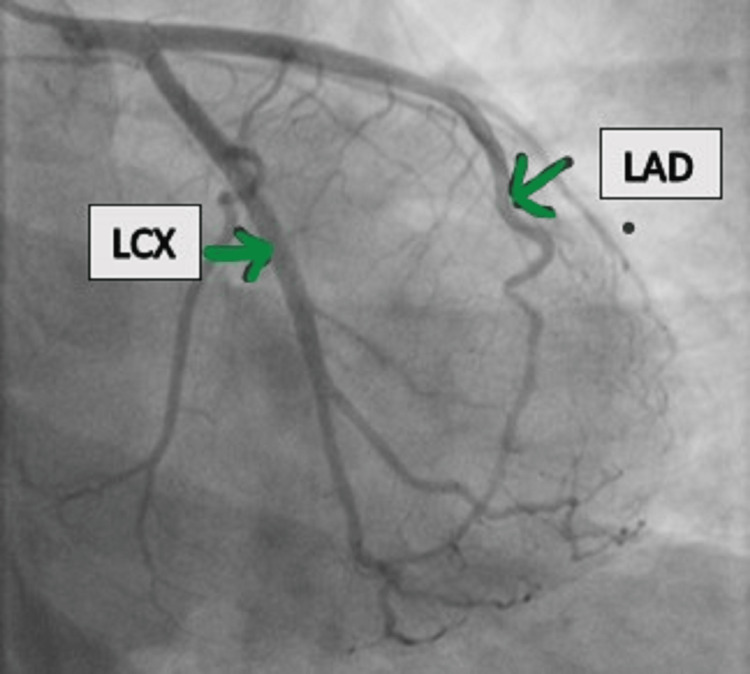
AP caudal view of normal LAD AP, anteroposterior; LAD, left anterior descending artery; LCX, left circumflex artery

**Figure 3:**
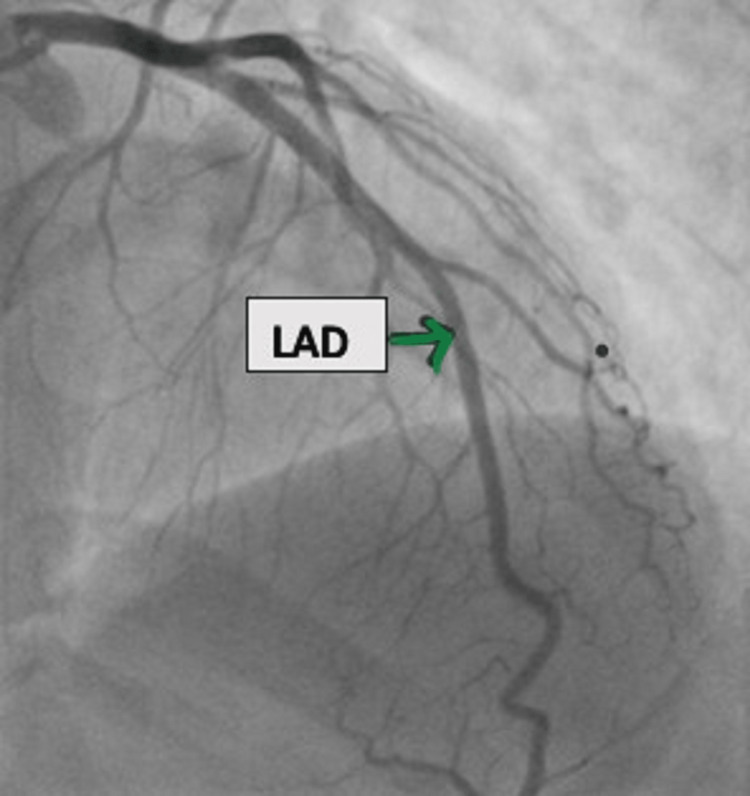
AP cranial view of LAD AP, anterioposterior; LAD, left anterior descending artery

**Figure 4:**
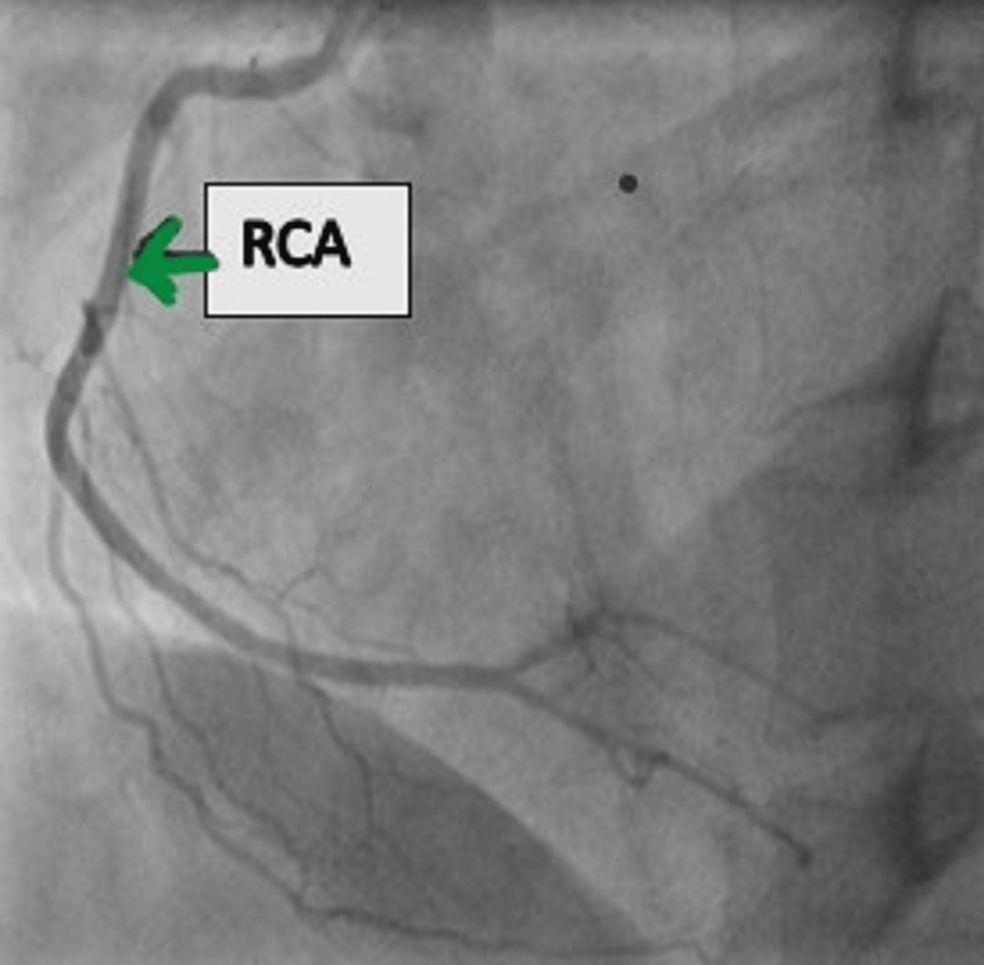
LOA view of normal RCA RCA, right coronary artery; LOA, left anterior oblique

Repeat bedside 2D echocardiography (ECHO) revealed a fresh hypokinetic basal inferior wall with an LVEF of 45%. Her troponin I was elevated to 0.83 ng/dL. Without delay, the patient was taken for coronary angiography, which revealed normal coronaries (Figures 2-4).

Routine blood tests showed an elevated total leucocyte count of 32,500 mm^3^ of blood; however, other parameters were normal, including serum creatinine. We continued supportive treatment for her. Over the next few days, she gradually improved both symptomatically and hemodynamically, oxygen and inotropes were tapered off, and the dose of diuretics was reduced. She was discharged after five days in stable condition. At the time of discharge, her LVEF improved to 50%. On the first follow-up after two weeks, her ECHO revealed a completely recovered left ventricular (LV) function with an LVEF of 65%. Over 10 weeks of follow-up, she is asymptomatic and off any medication except for a small dose of beta blocker, which we plan to taper off soon. 

## Discussion

TTC, also known as “stress cardiomyopathy” or “broken heart syndrome,” is a temporary and reversible heart condition mimicking ACS but with normal coronaries. It typically occurs following intense emotional and mental stress [1]. It commonly presents as LV apical ballooning with a normal, narrow base (thereby mimicking the Japanese vessel takotsubo that Japanese people use to trap octopus) [9]. However, reverse TTC is also a well-recognized entity now, where basal segments are more affected than apical segments, as in our case [10]. TTC is a diagnosis of exclusion, whereby one needs to rule out more common conditions with similar clinical presentation and investigation parameters, such as obstructive coronary artery disease and myocarditis, before making a diagnosis of TTC [9]. Revised Mayo Clinic diagnostic criteria are helpful in clinching the diagnosis, which include the following points [11]: transient dyskinesis, akinesia or hypokinesia of the LV midsegments, with or without involvement of apical segments, regional wall motion abnormalities beyond a single epicardial vascular distribution, absence of obstructive coronary artery disease or acute plaque rupture, new electrocardiographic abnormalities or modest troponin elevation, and absence of pheochromocytoma and myocarditis. Our patient fulfilled all these criteria.

TTC occurs predominantly in post-menopausal women soon after exposure to sudden unexpected emotional or physical stress. However, no age group is immune. A literature search reveals cases ranging from infancy to 98 years [12]. There is a striking female preponderance. Catecholamine surge is widely accepted as an underlying predominant factor with resultant myocardial stunning, multivessel coronary artery spasm, impaired fatty acid metabolism, and endothelial dysfunction [10]. A study of the literature shows that apart from emotional and psychological stress, there are several cases where external factors contribute to TTC, such as surgery, fracture, vaginal delivery, anti-cancer drugs, and amiodarone [13]. Exogenous catecholamine administration is also a well-recognized trigger.

In our patient, who has otherwise been physically as well as mentally fit, an anaphylactic reaction to the COVID-19 vaccine with severe hypotension, acute bronchospasm, and cerebral hypoperfusion with resultant transient loss of vision, everything combined was enough to result in a deja-vu situation with an impending sense of doom. This caused extreme anxiety and agitation, in itself enough to cause an acute surge of catecholamines and precipitate TTC in susceptible individuals. To compound the matter, she was administered exogenous catecholamines. Both these factors must have combined to result in her condition. Her ECG revealed ST-T changes with QT prolongation. A chest X-ray showed pulmonary edema, which resolved later. Her ECHO showed RWMA with a lowering of LVEF. Her troponin level was also elevated, but coronary angiography was normal, clinching the diagnosis of takotsubo. Our patient recovered completely, like other TTC patients, further strengthening our accurate diagnosis.

Finally, although all the currently available COVID-19 vaccines are safe, the incidence of anaphylaxis is significantly lower in COVISHIELD compared to Pfizer. This is because of the presence of the potential allergen PEG-2000 present in the Pfizer vaccine but not in the COVISHIELD [14]. In a phase 3 clinical trial, where 12,000 patients were administered an active vaccine, none of them had any anaphylaxis. In real-life scenarios too, the incidence of anaphylaxis is significantly low, less than one in 10,000. Our patient underlines the fact that although rare, potentially life-threatening anaphylaxis is very much a possibility, and all vaccination centers need to be well-equipped to handle any such emergency.

## Conclusions

We report a first-documented case of TTC resulting from anaphylaxis to COVISHIELD vaccine, which is a potentially reversible condition provided it is recognized and treated on time. Our study also highlights the fact that although rare, anaphylaxis can still happen, and all vaccination centers must be well-equipped to deal with these emergencies.
